# Effectiveness of optic nerve sheath fenestration in preserving vision in idiopathic intracranial hypertension: an updated meta-analysis and systematic review

**DOI:** 10.1007/s00701-024-06345-y

**Published:** 2024-11-25

**Authors:** Kacper Prokop, Aleksandra Opęchowska, Andrzej Sieśkiewicz, Łukasz Lisowski, Zenon Mariak, Tomasz Łysoń

**Affiliations:** 1 Department of Neurosurgery, Medical University of Bialystok, Bialystok, Poland; 2 Department of Otolaryngology, Medical University of Bialystok, Bialystok, Poland; 3 Department of Ophthalmology, Medical University of Bialystok, Bialystok, Poland

**Keywords:** Meta-analysis, Idiopathic intracranial hypertension, Benign intracranial hypertension, Pseudotumor cerebri, Optic nerve sheath fenestration, Optic nerve sheath decompression

## Abstract

**Background:**

This study aims to evaluate the effectiveness of Optic Nerve Sheath Fenestration (ONSF) in improving visual outcomes in patients with Idiopathic Intracranial Hypertension (IIH).

**Methods:**

A meta-analysis was conducted using data from 19 studies, totaling 1159 observations. The main assessed outcomes after ONSF surgery were: the improvement in visual acuity, the improvement in visual fields and reduction in papilledema. We performed separate analyses to distinguish between outcomes using different surgical approaches. Comprehensive literature searches were conducted in the Ovid MEDLINE(R) and SCOPUS databases, following PRISMA guidelines. Statistical analyses employed a Generalized Linear Mixed Model (GLMM) to integrate proportion data, with heterogeneity assessed via I-squared and H statistics. Publication bias was evaluated using funnel plots, Egger's test, and Peters' test.

**Results:**

The analysis revealed that ONSF significantly improved visual acuity in 41.09% of patients, and visual fields in 76.34% of cases. The transconjunctival approach demonstrated higher success rates for visual field improvement. A high improvement rate of 97% was observed in reducing optic disc swelling. Significant heterogeneity was noted, particularly in visual acuity (I^2^ = 92.1%) and visual field improvements (I^2^ = 73.8%), reflecting variability in surgical techniques and patient demographics. Publication bias assessments indicated no significant bias for visual acuity and visual field outcomes, although potential bias was detected for papilledema reduction.

**Conclusions:**

This meta-analysis confirms that ONSF is effective in improving visual outcomes for IIH patients, especially when using the transconjunctival approach. Early surgical intervention is crucial for optimal results, principally in preventing advanced ischemic damage. Despite high success rates, observed heterogeneity highlights the need for standardized surgical techniques and further investigation into patient-specific factors influencing outcomes. Addressing potential publication bias and conducting more rigorous studies will enhance the reliability of future meta-analyses. Upcoming research in ONSF efficacy is needed to refine surgical practices and optimize patient care.

## Introduction

Idiopathic intracranial hypertension (IIH), previously known as "pseudotumor cerebri" or "benign intracranial hypertension," is manifested mainly by vision disturbances, headaches, and dizziness, due to increased intracranial pressure (ICP). The condition predominantly affects women of reproductive age and is strongly associated with obesity [23, 30], though its etiopathogenesis is vaguely understood. Most patients experience usually bilateral and symmetric papilledema, which can lead to vision loss in severe cases. Diagnosis is guided by the modified Dandy criteria, updated by Friedman. These criteria include: a) signs of increased ICP, papilledema, or abducens palsy; b) normal neurological exam excluding cranial nerve abnormalities; c) normal contrast and non-contrast MRI or contrast CT; d) normal cerebrospinal fluid (CSF) composition; and e) elevated lumbar puncture opening pressure.

Management of IIH aims to reduce elevated ICP. Initial treatment typically involves non-surgical methods like weight loss and diuretics as first-line therapy. However, non-surgical options remain ineffective in about 25% of patients, necessitating surgical interventions such as optic nerve sheath fenestration (ONSF), CSF diversion, venous sinus stenting, or bariatric surgery [23]. ONSF is a clinically recognized method for managing IIH. It involves creating an opening in the optic nerve sheath to reduce CSF pressure by releasing CSF from the subarachnoid space in the orbit. This procedure aims at preserving vision, threatened by elevated ICP rather than treating the underlying cause of IIH. Additionally, ONSF is associated with low risk of serious complications [12, 36], especially when using minimally invasive endoscopic transconjunctival techniques [27].

Several studies have reported the clinical efficacy of ONSF in relieving IIH symptoms, but comprehensive assessments of its exact efficacy in vision preservation are lacking. The previous meta-analyses provided by Scherman et al. [43] and by Satti et al. [42] addressed the effectiveness of various surgical interventions for IIH, including ONSF, venous sinus stenting and CSF diversion and provide valuable insights into the subject of invasive treatment of IIH. However, Scherman's analysis did not include the evaluation of visual fields, which we believe is critical for assessing the full scope of visual function during the treatment. Additionally, Satti's work, though comprehensive, with time became somewhat outdated and does not incorporate the latest data and advancements. Therefore, to address these gaps, we decided to conduct an updated meta-analysis, focusing exclusively on visual outcomes: visual fields, visual acuity, and optic disc swelling, since we trust these are crucial for an objective assessment of ONSF's effectiveness. Therefore, we evaluated more recent, postoperative data from 19 studies spanning 33 years, covering a total of 1159 observations, with separate analyses to distinguish between outcomes using different surgical approaches. The results provide a comprehensive and current evaluation of the clinical effectiveness of ONSF and thus enable the synthesis of evidence on the clinical outcomes of ONSF surgery, particularly its role in maintaining proper vision in IIH patients.

## Materials and methods

The primary aim of this analysis was to determine the collective effect sizes for three key surgical outcomes: improvement in visual acuity, enhancement of visual field, and post-operative reduction in optic disc swelling. Only the side where the ONSF was performed was evaluated. Due to variable assessment methods, outcomes were categorized as improvement or no improvement in visual acuity, visual field, and papilledema. Additionally, we performed separate analyses to distinguish between outcomes using different approaches for fenestration. A meta-analysis of studies, selected according to the PRISMA methodology, used the PICOTS framework to guide the research:

- Patient Population (P): Adult patients diagnosed with IIH,
- Intervention (I): Optic Nerve Sheath Fenestration (ONSF) surgery,
- Comparator (C): Preoperative condition of patients,
- Outcomes (O): Improvement in visual acuity, visual fields, and reduction in optic disc swelling,
- Time (T): Earliest follow-up data from each study for consistency,
- Setting (S): Various clinical settings in multiple healthcare facilities.

### Data sources and search strategy

The literature search was conducted using the Ovid MEDLINE(R) database (1946 to June 12, 2024) and the SCOPUS database (1974—June 12, 2024). The search strategy followed the guidelines outlined by the Preferred Reporting Items for Systematic Reviews and Meta-Analyses (PRISMA) [37]. This study was registered in the International Prospective Register of Systematic Reviews PROSPERO (CRD42024557743).

### Medline search strategy (via OVID)

The search terms and strategy implemented in Medline are detailed in Table 1. The search focused on combinations of terms related to IIH, ONSF, and visual outcomes, using Medical Subject Headings (MeSH) and keyword searches. The strategy included:

1. Condition Terms:
  Idiopathic intracranial hypertension
  Pseudotumor cerebri
  Benign intracranial hypertension
2. Intervention Terms:
  Optic nerve sheath fenestration
  Optic nerve sheath decompression
  Optic nerve decompression
3. Outcome Terms:
  Visual disturbances
  Visual impairment
  Vision loss
  Blindness
  Vision deterioration

**Table 1:** MEDLINE search strategy (via OVID)

1 exp idiopathic intracranial hypertension/
2 exp pseudotumor cerebri/
3 exp benign intracranial hypertension
4 1 or 2 or 3
5 optic nerve* sheath* fenestratnion*.mp
6 optic nerve* sheath* decompression*.mp
7 optic nerve* decompression*.mp
8 5 or 6 or 7
9 visual disturbance*.mp
10 visual impairment.mp
11 vision loss.mp
12 visual loss.mp
13 blind*.mp
14 vision.mp
15. visual.mp
16 visual field*.mp
17 ision deterioration.mp
18 9 or 10 or 11 or 12 or 13 or 14 or 15 or 16 or 17
19 4 and 8 and 18

The search combined these terms using Boolean operators to maximize the retrieval of relevant studies (Table 1 provides the detailed query syntax).

### Scopus search strategy

The search in SCOPUS was conducted using the TITLE-ABS-KEY field to capture terms within the title, abstract, and keywords of the publications. The search terms mirrored those used in the Medline search, ensuring comprehensive coverage across databases Table 2.

**Table 2:** SCOPUS search strategy

(TITLE-ABS-KEY ( "idiopathic intracranial hypertension" OR "pseudotumor cerebri" OR "benign intracranial hypertension") AND TITLE-ABS-KEY ( "optic* nerve* sheath* fenestration*" OR "optic* nerve* sheath* decompression*" OR "optic* nerve* decompression*") AND TITLE-ABS-KEY ( "visual disturbance*" OR "visual impairment*" OR "vision loss" OR "visual loss" OR "blind*" OR "vision" OR "vis*" OR "visual" OR "visual field*" OR "vision deterioration"))	

### Study selection process

**Fig. 1:**
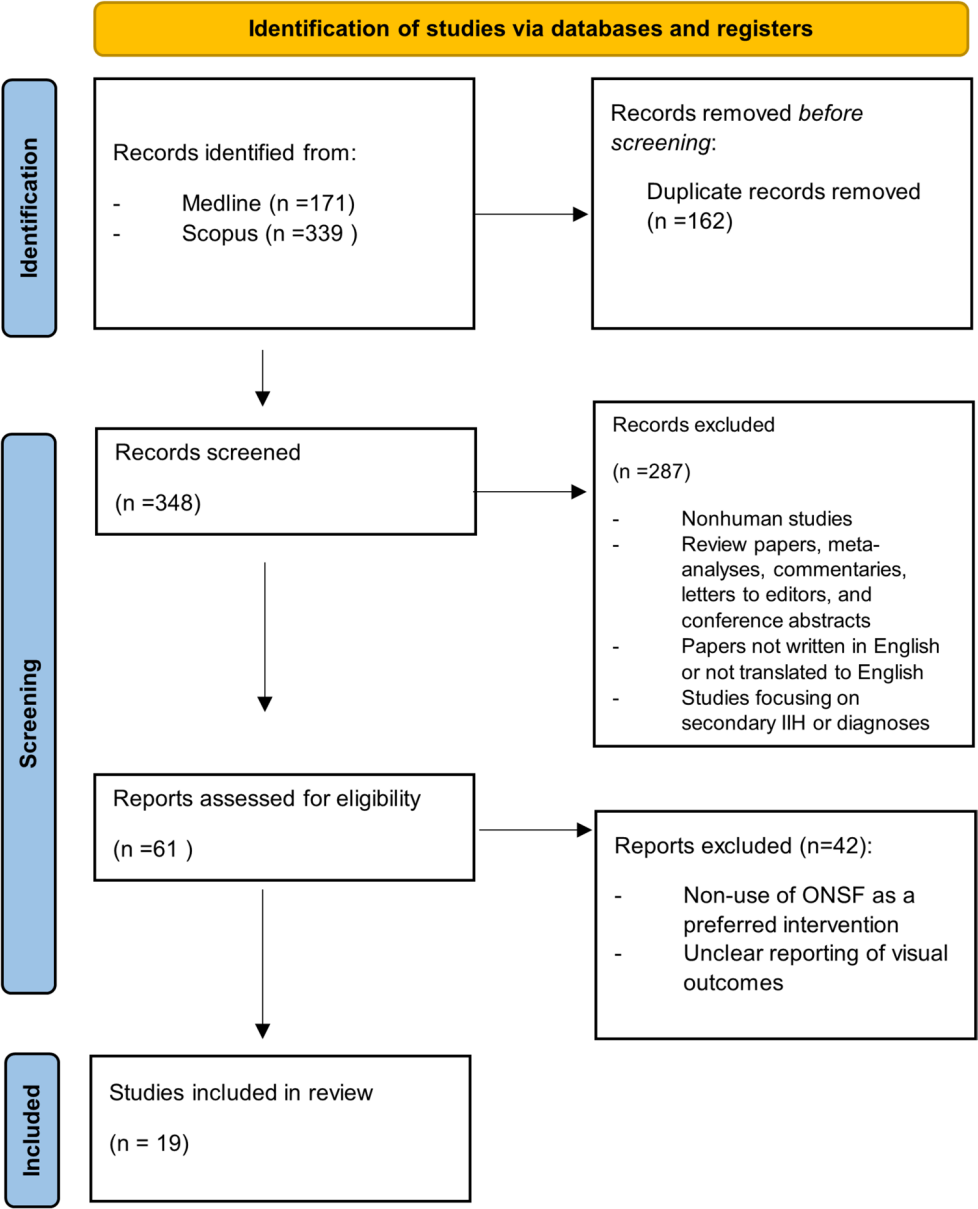
The phases of the study selection process

The study selection process was conducted in three main phases: identification, screening, and eligibility assessment, following the PRISMA flow diagram (Fig. 1) [37].

### Identification

A total of 510 records were identified through database searches: 171 from Medline and 339 from SCOPUS. The articles were reviewed using Rayyan software. After removing 162 duplicate records, 348 unique records were assessed for relevance by two independent reviewers (K.P. and A.O.)

### Screening

During the screening phase, the titles and abstracts of the 348 records were reviewed to exclude irrelevant studies. This phase led to the exclusion of 287 records based on predefined criteria:

- Non-human studies,
- Review papers, meta-analyses, commentaries, letters to editors, and conference abstracts,
- Papers not written in English or not translated into English,
- Studies focusing on secondary IIH or diagnoses other than IIH,
- Studies in which ONSF has not been chosen as a treatment modality,
- Non-extractable data,

### Eligibility assessment

The full texts of the remaining 61 reports were assessed for eligibility. This phase aimed to ensure that only studies meeting all inclusion criteria were considered for the meta-analysis. The specific inclusion criteria were:

- Studies involving human subjects diagnosed with primary IIH,
- Use of ONSF as an intervention of choice,
- Clear reporting of visual outcomes,

Out of the 61 reports, 42 were excluded based on these criteria, resulting in 19 studies [1, 7, 9, 14, 15, 17, 20, 22, 24, 25, 29, 31, 34, 35, 40, 45, 46, 47, 52] being included in the final meta-analysis.

### Data extraction and quality assessment

Data extraction captured essential study characteristics, patient demographics, intervention details, and outcomes using a standardized form. Study quality was assessed with the Newcastle–Ottawa Scale (NOS) for cohort studies. Discrepancies in data extraction and quality assessment were resolved through reviewer discussions.

### Statistical analysis

In our study, the alpha level for statistical significance was set at α = 0.05. The meta-analysis used a Generalized Linear Mixed Model (GLMM) to integrate proportion data across selected studies, computing pooled proportions using logit transformation and back-transformation for reporting [19, 44, 48, 53]. Confidence intervals for individual study results were derived using the normal approximation method [2]. Variance and standard deviation of true effect sizes (τ^2^ and τ) were estimated with the maximum likelihood approach [50], while I-squared (I^2^) and H statistics measured heterogeneity among studies [21]. The presence of heterogeneity was tested using the Wald and Likelihood Ratio Tests (LRT).

Publication bias was assessed with multiple methods. A funnel plot provided a visual inspection for asymmetries, suggesting potential bias. Egger's test quantified funnel plot asymmetry by regressing standardized effect estimates against their precision [11]. Peters' test examined the relationship between effect estimates and their variance (33).

### Characteristics of the statistical tool

*Analyses were conducted using the R Statistical language (version 4.3.1; R Core Team, 2023* [41]*) on Windows 10 pro 64 bit (build 19045), using the packages meta (version 6.5.0* [5]*;), Matrix (version 1.6.1.1* [6]*;), numDeriv (version 2016.8.1.1* [13]*;) lubridate (version 1.9.3* [16]*;), dmetar (version 0.1.0* [18]*;), report (version 0.5.7* [28]*;), tibble (version 3.2.1* [33]*;), metafor (version 4.4.0* [51]*;), metadat (version 1.2.0* [54]*;), ggplot2 (version 3.4.4* [55]*;), dplyr (version 1.1.3* [56]*;), purrr (version 1.0.2* [57]*;), and tidyr (version 1.3.0* [58]*;).*

## Results

### Estimating the pooled effect of the proportion for improved visual acuity post-surgery

**Fig. 2:**
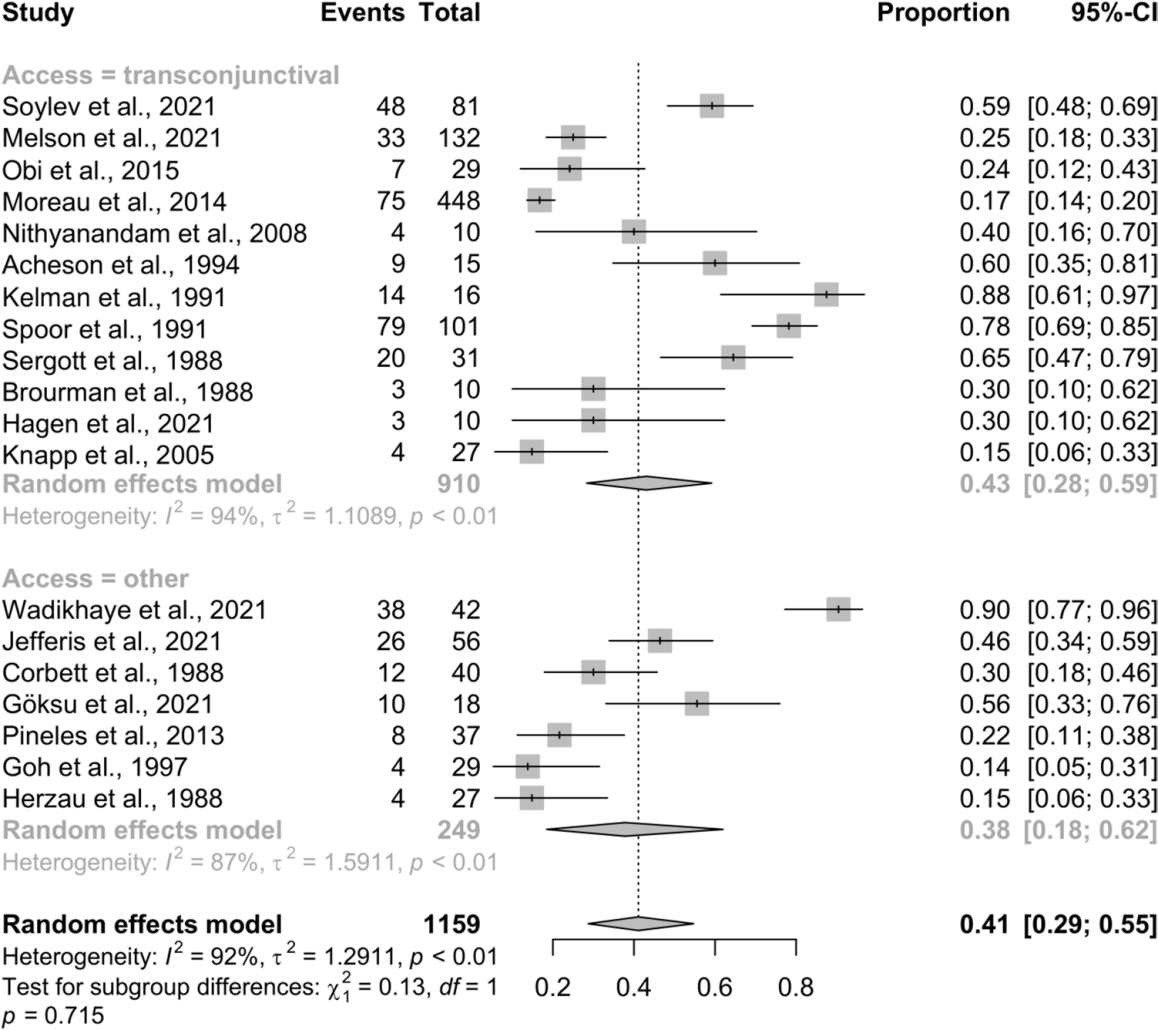
Pooled proportion of improved visual acuity post-surgery: a forest plot subgroup analysis by access factor

This meta-analysis included data from 19 studies with 1,159 observations, documenting 401 events of improved visual acuity post-surgery. Female proportions ranged from 0.64 to 1.00 (Fig. 2). In 63% of cases, surgery used the transconjunctival approach, while 37% used a different method. Using a random effects model, the pooled proportion of patients with improved visual acuity was 0.4109 (95% CI: 0.29 to 0.55), indicating about 41.09% showed improvement.

Significant heterogeneity was observed among the studies. The τ^2^ value was 1.29, with τ (the standard deviation) at 1.14. The I^2^ statistic was 92.1% (CI: 89.1% to 94.3%), and the H statistic was 3.56 (CI: 3.03 to 4.18), both indicating substantial variability in effect sizes. Heterogeneity tests, including the Wald test and LRT, confirmed significant variability (p < 0.001). By approach method, there were no significant differences in improvement proportions (*p = *0.715).

**Fig. 3:**
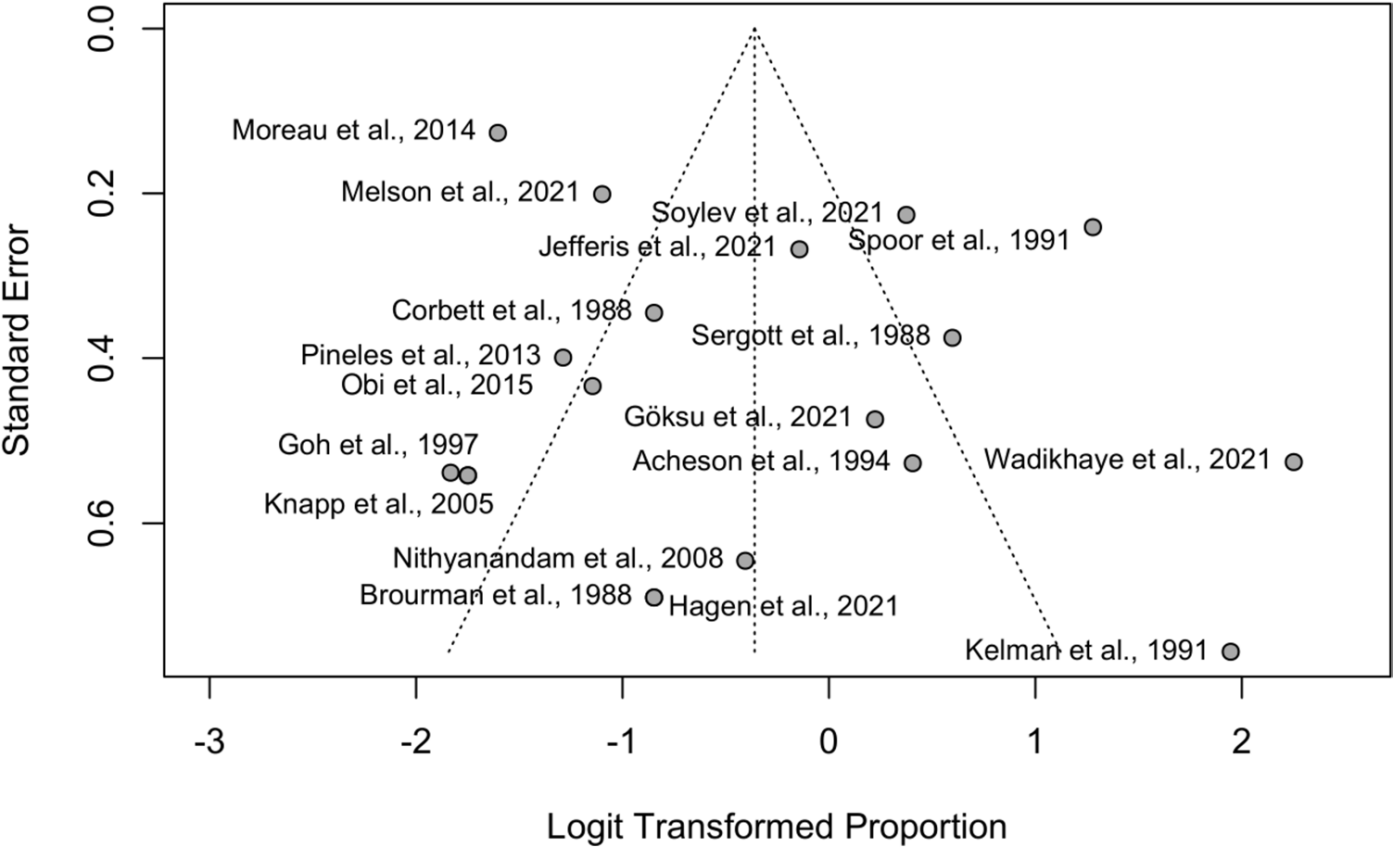
Funnel plot of logit-transformed proportion estimates versus standard error for assessing publication bias in studies on improved visual acuity post-surgery

The funnel plot in Fig. 3 exhibits effect estimates from individual studies against study precision (standard error). Logit transformation of proportions was used to normalize effect sizes. The vertical line represents the pooled estimate of the logit-transformed proportions, serving as a reference for individual study estimates. The diagonal 'funnel lines' depict the expected range of study distribution in the absence of publication bias, ideally showing a symmetric scatter around the vertical line.

The funnel plot in Fig. 3 demonstrates a symmetric distribution of studies around the central vertical line, suggesting no publication bias. The even scatter of studies indicates that smaller and larger studies report similar effect sizes, reinforcing the reliability of the meta-analysis. Both Egger's and Peters' tests confirmed this visual assessment with non-significant p-values (*p = *0.173 and *p = *0.330, respectively), indicating the findings are robust and not influenced by selective publication of favorable outcomes.

### Estimating the pooled effect of the proportion of visual field improvement post-surgery

**Fig. 4:**
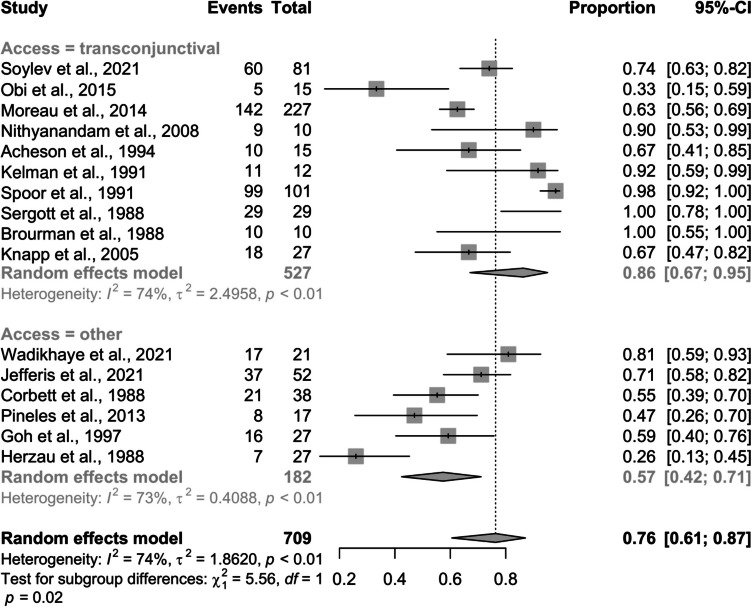
Pooled proportion of visual field improvement post-surgery: A forest plot subgroup analysis by access factor

The meta-analysis estimated the pooled effect of visual field improvement post-surgery using data from 16 studies (709 observations, 499 events). A random effects model estimated a pooled improvement proportion of 0.7634 (95% CI: 0.61 to 0.87), indicating substantial improvement (Fig. 4). The surgery was performed using the transconjunctival method in 63% of cases and other methods in 37%.

Significant heterogeneity was detected, with an I^2^ value of 73.8% and p < 0.001, suggesting that 74% of variability is due to genuine differences between studies. The τ^2^ value was 1.86, and τ was 1.36. Subgroup analysis showed differing effects based on surgical approach. Transconjunctival approach reported a higher improvement proportion (0.86, CI: 0.67 to 0.95) with high heterogeneity (τ^2^ = 2.50, I^2^ = 74.3%). Other methods showed a lower proportion (0.57, CI: 0.42 to 0.71) and lower heterogeneity (τ^2^ = 0.41, I^2^ = 72.8%). The test for subgroup differences was significant (*p = *0.018).

**Fig. 5:**
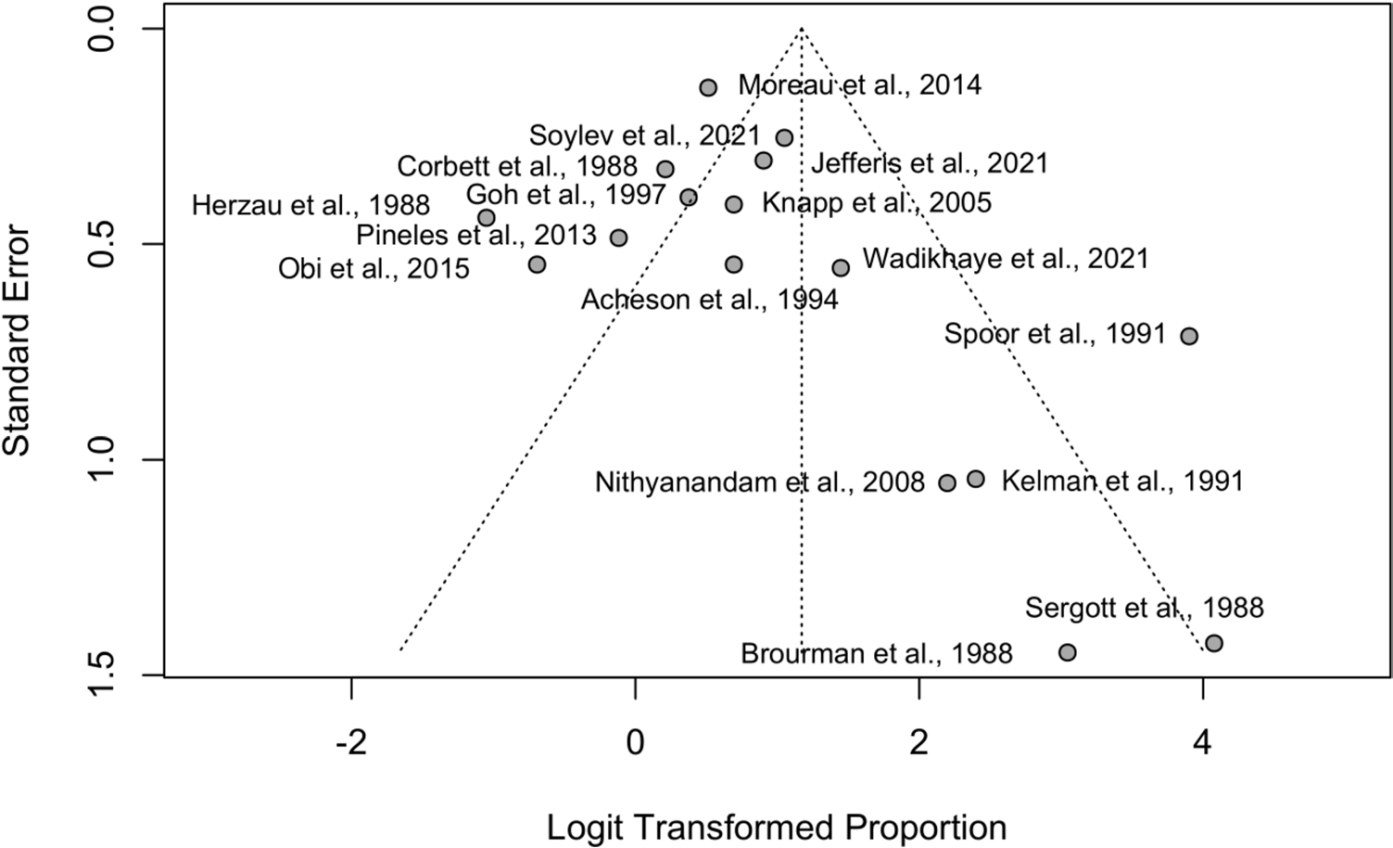
Funnel plot of logit-transformed proportion estimates versus standard error for assessing publication bias in studies on visual field improvement post-surgery

The funnel plot analysis in Fig. 5 demonstrates studies with smaller standard errors on the left and those with larger standard errors on the right. Despite this distribution, Egger's test (*p = *0.177) does not indicate significant publication bias. Thus, the asymmetry in the funnel plot is not statistically significant, suggesting that smaller studies' effect sizes do not systematically diverge, indicating no publication bias.

Peters' test (*p = *0.633) further confirms the absence of significant publication bias, reinforcing Egger's test results. These findings indicate no significant asymmetry concerning study size, affirming the meta-analysis's integrity and reliability. Although initial visual assessments suggested otherwise, the conclusions of the meta-analysis remain strong and well-substantiated by the data.

### Estimating the pooled effect of the proportion for optic disc swelling improvement post-surgery

**Fig. 6:**
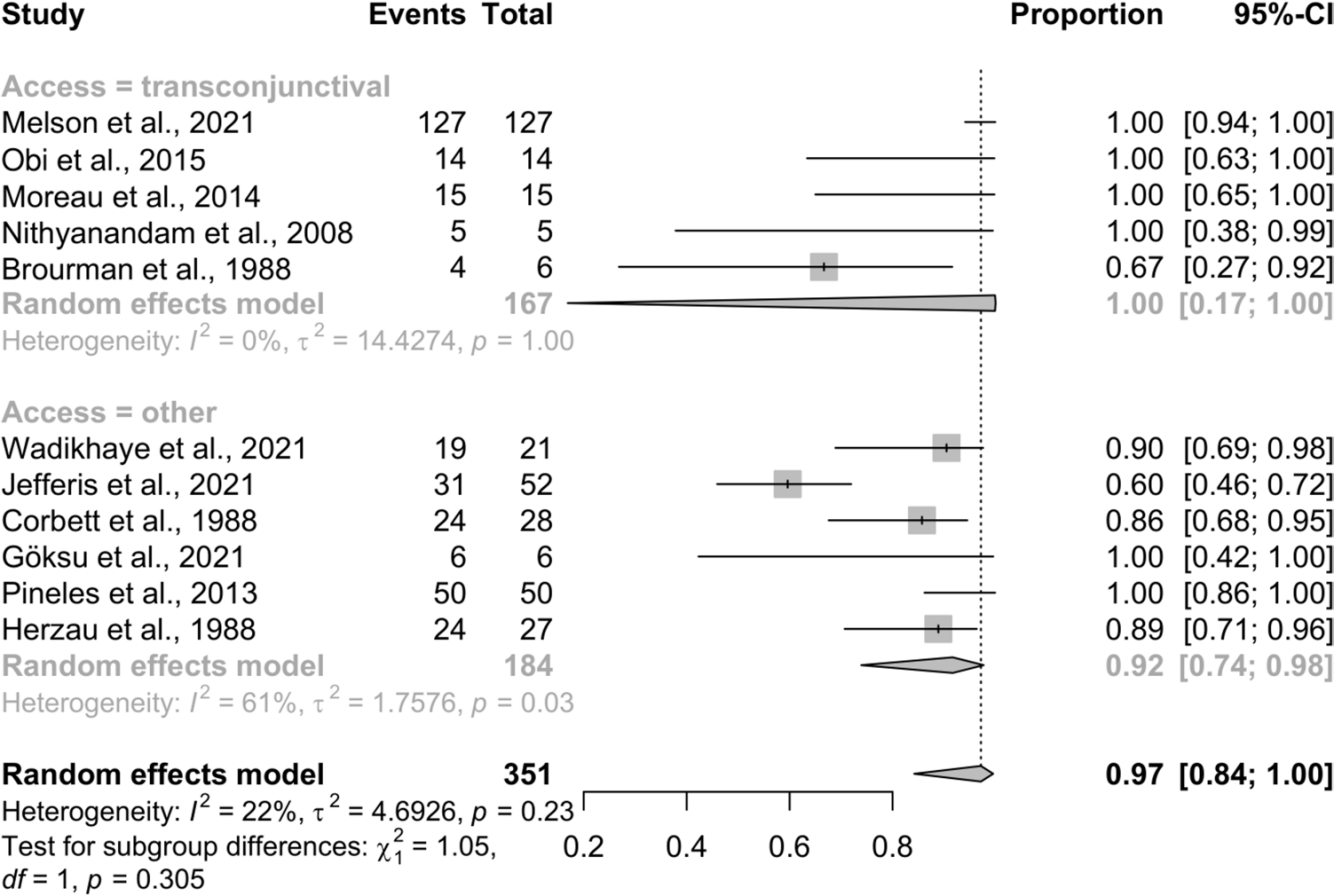
Pooled proportion of optic disc swelling improvement post-surgery: a forest plot subgroup analysis by access factor

The meta-analysis evaluated 11 studies (351 observations, 319 events) to estimate the pooled effect of improvement in optic disc swelling post-surgery. Using a random effects model, the pooled effect proportion was 0.97 (CI: 0.84 to 1.00), indicating significant improvement in most cases (Fig. 6). The transconjunctival approach was used in 45.5% of surgeries, and other methods in 54.5%.

Heterogeneity analysis showed a low I^2^ value of 22.4%, suggesting modest variance due to heterogeneity. The Wald test showed no significant heterogeneity (*p = *0.231), while the LRT did (p < 0.001), indicating some underlying but not uniformly detectable heterogeneity.

Subgroup analysis based on surgical approach showed transconjunctival approach with an improvement proportion of 0.9990 (CI: 0.17 to 1.000) and high heterogeneity (τ^2^ = 14.43). Other approaches had a lower improvement proportion of 0.92 (CI: 0.74 to 0.98) with moderate heterogeneity (τ^2^ = 1.76, I^2^ = 60.8%). The test for subgroup differences was not statistically significant (*p = *0.305), indicating that the type of surgical approach did not significantly influence improvement.

**Fig. 7:**
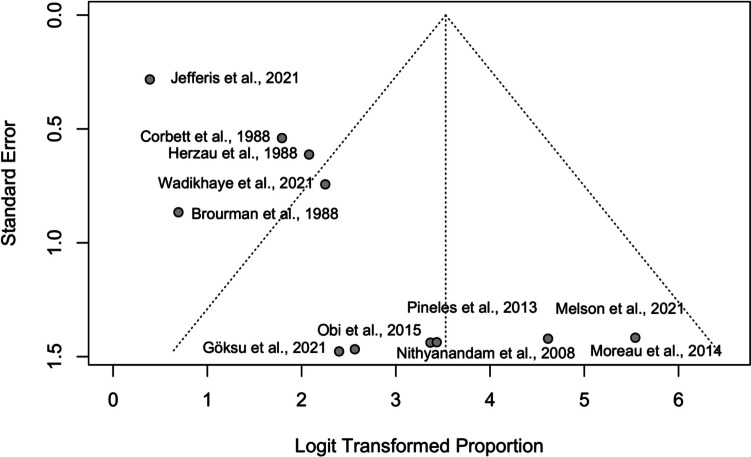
Funnel plot of logit-transformed proportion estimates versus standard error for assessing publication bias in studies evaluating improvement in optic disc swelling after surgery

The analysis of publication bias in the meta-analysis on optic disc swelling post-surgery reveals differing results from Egger's and Peters' tests. Egger's test indicates significant publication bias (*p = *0.001), with a small standard error (0.56) and a negative intercept (-0.21), suggesting smaller studies might report higher effects, as seen in Fig. 7. Conversely, Peters' test found no evidence of publication bias (*p = *0.777), with a large standard error (12.52) indicating less precise estimation. This discrepancy might be due to the smaller degrees of freedom in Peters' test.

The discrepancy between these tests could be attributed to their methodological differences. Egger's test is generally more sensitive to asymmetries caused by smaller studies, while Peters' test might be less sensitive to such effects due to its reliance on the total sample size.

### Used surgical approaches

To provide a comprehensive insight into the surgical approaches used in the analysed studies, we present a table summarizing different surgical methods and number of patients in each group (Table 3).

**Table 3:** Summary of used surgical approaches

	Study	Approach	Extensive description	No. of patients	Instrumentation	Muscle disinsertion
1	Soylev et al., [46]	Medial transconjunctival	Medial limbal peritomy	56	Non-specified	+
2	Melson et al., [29]	Medial transconjunctival	A 10 mm curvilinear incision was made trough the conjunctiva in the superonasal fornix	66	Microscope	-
3	Obi et al., [35]	Medial transconjunctival	An eye speculum was inserted to maintain an adequately sized palpebral aperture. A 270° conjunctival peritomy was performed incorporating the medial and superior limbus	14	Non-specified	+
4	Moreau et al., [31]	Medial transconjunctival	-	236	Non-specified	+
5	Nithyanandam et al., [34]	Medial transconjunctival	A 270° medial peritomy	5	Microscope	+
6	Acheson et al., [1]	Medial transconjunctival	-	11	Microscope	+
7	Kelman et al., [24]	Posterolateral transconjunctival	A 180° conjunctival incision, 4 mm posterior to the limbus	12	Microscope	+
8	Spoor et al., [47]	Medial transconjunctival	-	53	Microscope	+
9	Sergott et al., [45]	Posterior transconjunctival	A 270° conjunctival incision was made 4 mm posterior to the corneoscleral junction	23	Microscope	+
10	Brourman et al., [7]	Medial transconjunctival	-	6	Non-specified	+
11	Hagen et al., [17]	Superonasal transconjunctival	The conjunctiva was opened with scissors in the superonasal quadrant 3 mm from and parallel to the limbus	10	Microscope	-
12	Knapp et al., [25]	Medial transconjunctival	-	13	Non-specified	+
13	Wadikhaye et al., [52]	Fronto-temporo-sphenoidotomy	A 270° optic canal decompression via pterional craniotomy that was followed by extradural clinoidectomy and optic foramen bony decompression using an ultrasonic aspirator	21	Microscope	-
14	Jefferis et al., [22]	Supero-medial eyelid skin crease approach	An incision was made along the supero-medial eyelid skin crease and orbicularis followed by dissection between orbicularis and septum almost to the orbital rim	30	Microscope	-
15	Corbett et al., [9]	Lateral or combined lateral and medial orbitotomy	-	28	Non-specified	Non-specified
16	Göksu et al., [15]	Transnasal endoscopic	Completing up to 180◦ of bony decompression of the optic canal (medial and inferior wall)	9	Endoscope	-
17	Pineles et al., [40]	Non-specified	-	37	Non-specified	Non-specified
18	Goh et al., [14]	Medial and lateral orbitotomies	-	19	Non-specified	Non-specified
19	Herzau et al., [20]	Non-specified	-	15	Non-specified	Non-specified

## Discussion

### Improvement in visual acuity

The pooled proportion of patients experiencing improved visual acuity post-surgery was estimated at 41.09%, suggesting a positive outcome for a significant portion of patients. However, the high degree of heterogeneity (I^2^ = 92.1%) indicates considerable variability in outcomes across different studies. This variability could be attributed to differences in surgical techniques, patient populations, or measurement methods. The lack of significant difference between surgical approaches (*p = *0.715) implies that the improvement in visual acuity is relatively consistent regardless of the approach used. This is encouraging for clinical practitioners as it suggests flexibility in choosing surgical techniques based on patient-specific factors without compromising outcomes.

Wadikhaye et al. [52] reported a remarkable 90.47% improvement in visual acuity among patients undergoing transcranial ONSF using an ultrasonic aspirator. Similarly, Moreau et al. [31] found that visual acuity remained stable or improved in 94.4% of eyes following the medial transconjunctival ONSD approach. This high rate of success was also echoed by Söylev Bajin et al. [46], who observed substantial improvements in best-corrected visual acuity (BCVA) in 62% of operated eyes.

Jefferis et al. [22] documented that visual acuity improved in 46.4% of eyes and was stable or improved in 92.9% of eyes post-ONSF using the supero-medial eyelid skin crease approach, which minimalized complications related to muscle disinsertion. The consistency across these studies highlights the efficacy of ONSF and ONSD in enhancing visual acuity for patients with IIH, making these surgical interventions crucial for preventing vision loss in this population. Moreover, in the meta-analysis by Scherman et al. [43] there was a 44% improvement in visual acuity, which is consistent with our results. Satti et al. [42] reported that 59% of eyes improved in visual acuity.

### Improvement in visual field

The analysis showed a substantial pooled proportion of visual field improvement post-surgery (76.34%). This high proportion reflects the overall efficacy of surgical interventions in enhancing visual fields for patients. Similar to visual acuity, significant heterogeneity was observed (I^2^ = 73.8%). Subgroup analysis revealed that transconjunctival approaches yielded higher improvement proportions (0.86) compared to other methods (0.57), with a significant difference (*p = *0.018). This finding suggests that the transconjunctival approach may be more effective for visual field improvement, warranting further investigation into optimizing surgical techniques to enhance patient outcomes.

Wadikhaye et al. [52] reported an 80.95% improvement in visual fields in patients treated with transcranial ONSF. Moreau et al. [31] observed that visual fields remained stable or improved in 95.9% of eyes following the medial transconjunctival approach to ONSD. Söylev Bajin et al. [46] further confirmed these findings, with 79% of operated eyes showing visual field improvements. A meta-analysis by Satti et al. [42] reported an improvement rate of 68% in the visual fields.

These findings collectively emphasize the substantial benefits of ONSF in preserving and enhancing visual fields, which are crucial for the overall visual function of patients with IIH.

### Improvement in optic disc swelling

The improvement in optic disc swelling post-surgery was highly significant, with a pooled proportion of 97%. This near-perfect improvement rate underscores the effectiveness of surgical interventions in addressing optic disc swelling. Heterogeneity was relatively low (I^2^ = 22.4%), indicating consistent results across studies. The subgroup analysis by surgical approach revealed that the transconjunctival method achieved an improvement rate of 0.999 (CI: 0.17 to 1.000) but showed high heterogenity (τ^2^ = 14.43). Other surgical techniques demonstrated a slightly lower improvement rate of 0.92 (CI: 0.74 to 0.98) with moderate heterogenity (τ^2^ = 1.76, I^2^ = 60.8%). The lack of significant differences between surgical approaches (*p = *0.305) suggests that various surgical techniques are equally effective in reducing optic disc swelling. These findings underscore the effectiveness of surgery in reducing optic disc swelling, with high success rates across different methods. The heterogeneity suggests that individual study characteristics or patient populations could influence the outcomes.

Wadikhaye et al. [52] observed a 90.47% improvement in fundus pictures, indicating significant resolution of papilledema following transcranial ONSF. Söylev Bajin et al. [46] also documented notable reductions in papilledema, with improvements observed in both operated and non-operated fellow eyes. Scherman et al. [43] and Satti et al. [42] reported a 77%, and 80% post-ONSF reduction in papilledema, respectively.

These studies consistently show that ONSF is effective in significantly reducing papilledema, which is essential for preventing further visual deterioration and improving overall patient outcomes in IIH.

### Comparative efficacy of different surgical approaches

Various surgical techniques have been compared, revealing differences in efficacy and complication rates. The transcranial approach with an ultrasonic aspirator, as described by Wadikhaye et al. [52], showed better visual outcomes compared to other methods. Moreau et al. [31] and Melson et al. [29] highlighted the safety and effectiveness of the medial and superonasal transconjunctival approaches, respectively, which were associated with reduced postoperative complications such as diplopia. In the meta-analysis conducted by Corecha Santos et al. [10] assessing the effectiveness of EONSD, the improvement rate for papilledema was 73% in both unilateral and bilateral EONSD groups, and the overall improvement rate for visual impairment was 88%. However, the author did not evaluate visual acuity and visual field separately.

### Transconjunctival approach in optic nerve sheath fenestration

The transconjunctival approach for ONSF has demonstrated clear advantages over more invasive procedures, such as lower complication rates, reduced tissue trauma, and shorter surgery times. Research by Vaidya et al. [49] indicated that this method effectively alleviates optic disc edema and remains safe postoperatively, even in patients with advanced visual impairment. Additionally, the transconjunctival technique allows for rapid procedure completion, with bilateral surgeries taking around 25–30 min. Supporting this, Melson et al. [29] found that the superonasal transconjunctival approach is typically completed within 50 min and has minimal postoperative complications.

Jefferis et al. [22] highlighted the method’s effectiveness and safety, particularly through a superomedial eyelid incision, noting fewer complications compared to cerebrospinal fluid diversion surgeries. Also, the results of this meta-analysis support the positive personal experience of the authors with the endoscopic transconjunctival ONSF, performed since 2010, after the Pillai’s et al. report [39] demonstrating the feasibility of this approach on cadaver preparations. The approach, though technically demanding, does minimize surgical time and tissue disturbance by avoiding muscle disinsertion and eliminating the need for bone removal and eyelid suturing [26, 27].

Overall, these studies reinforce that transconjunctival ONSF provides a safer, more efficient alternative, significantly reducing tissue trauma and operative time.

### Techniques for incising the optic nerve sheath

Various techniques for incising the optic nerve sheath have been described. Some authors created windows of varying sizes, while others performed linear incisions, ranging from a single cut to several incisions. Söylev Bajin et al. [46] reported two linear incisions resulting in the leakage of cerebrospinal fluid (CSF). Nithyanandam et al. [34] described an incision of 4 to 5 mm in length in the avascular area of the anterior optic nerve sheath, approximately 2 mm posterior to the junction of the globe and the nerve. Sergott et al. [45] performed at least three longitudinal incisions, each measuring approximately 4 to 5 mm in length, 3 mm posterior to the junction of the optic nerve and the globe. Spoor et al. [47] reported multiple fenestrations in the optic nerve sheath, though the exact lengths of the incisions were not specified. They also noted that early failure (within several months) may result from inadequate fenestration or incision. This may be avoided by a more aggressive initial decompression with multiple fenestrations and incisions. Most authors reported creating a window in the optic nerve sheath measuring 3 × 2 mm [15, 22, 31, 52]; however, Jefferis et al. [22] described an initial cut of approximately 5 mm in length, followed by a second cut about 2 mm away, creating an opening of roughly 7 mm by 3 mm. There is no conclusive evidence regarding the optimal length of the optic nerve sheath incision. Further research is required to determine the most effective incision technique.

### Publication bias

The assessment of publication bias using funnel plots and statistical tests (Egger's and Peters' tests) provided mixed results. For visual acuity and visual field improvements, the analyses indicated no significant publication bias, reinforcing the reliability of the meta-analytic findings. However, for optic disc swelling, Egger's test suggested potential publication bias (*p = *0.001), while Peters' test did not (*p = *0.777). Considering these findings, while Egger's test suggests a potential concern regarding publication bias, the non-significant result from Peters' test provides a counterpoint, indicating that such bias may not be as pronounced when accounting for the overall sample sizes of the studies. This mixed evidence suggests the need for a cautious interpretation of the meta-analysis results.

### Missing data

In cases where data were missing from evaluated studies, only the available data were included in the meta-analysis. Studies with incomplete datasets were excluded from specific outcome analyses to maintain the integrity of the findings. The impact of this approach on the overall results was mitigated by the large number of observations included in the meta-analysis, allowing for robust conclusions despite some missing data.

### Implications for clinical practice

The findings from this meta-analysis have several implications for clinical practice. The demonstrated effectiveness of surgical interventions in improving visual outcomes and reducing optic disc swelling supports their continued use in clinical settings. The significant variability in outcomes, particularly for visual acuity and visual field improvements, underscores the need for individualized patient care and consideration of factors such as surgical technique and patient characteristics. The higher efficacy of the transconjunctival approach for visual field improvement suggests that it may be a preferable option in certain cases.

### Timing and predictors of outcomes

We were unable to perform a reliable analysis of the relationship between the timing of surgical intervention and the effectiveness of ONSF in preserving vision due to the lack of data in the reviewed studies. However, multiple authors emphasize the crucial importance of early intervention. The timing of surgery and the patient's preoperative visual function have been identified as significant predictors of postoperative outcomes. Numerous studies indicate that earlier surgical intervention correlates with better visual outcomes. For example, Hagen et al. [17] found that delayed surgery was associated with poorer visual field improvement, underscoring the necessity for timely intervention. Similarly, Pineles and Volpe [40] noted that patients with better preoperative visual function and earlier surgeries had improved outcomes. Furthermore, Söylev Bajin et al. [46] observed that patients who underwent ONSF during the early stages of papilledema, prior to the onset of optic atrophy, showed significant improvements in both visual acuity and visual fields. Chandrasekaran et al., [8] disclosed that delaying the procedure by more than six months from diagnosis significantly worsens visual field outcomes, underscoring the importance of timely surgery to improve prognosis for these patients. [8] Despite this, there is still no consensus on the ideal timing for ONSF. However, regular monitoring and timely intervention remain critical to prevent irreversible vision loss. [59] Hence, we present a summary of the available evidence on the importance of timely intervention (Table 4).

**Table 4:** Relationship between early surgery and clinical outcomes

	Study	Finding
1	Hagen et al., [17]	Prolonged time from diagnosis to surgery correlates negatively with perimetric mean deviation (PMD) improvement, with a correlation coefficient of r = − 0.78 and p < 0.01
2	Pineles et al., [40]	Shorter time to surgery was significantly associated with stabilization or improvement in visual acuity (VA) and visual field (VF) outcomes. The analysis demonstrated that better preoperative VA (*p = *0.01), color vision (*p = *0.002), and earlier surgical intervention (*p = *0.04) were predictors of more favorable outcomes
3	Soylev Bajin et al., [46]	It was indicated that earlier surgical intervention is associated with better clinical outcomes; however, precise numerical data or statistical values quantifying the impact of surgical delay on outcomes were not directly provided
4	Chandrasekaran et al., [8]	Delay to ONSF by more than 6 months following diagnosis of PTS was associated with poorer VF outcome (OR 0.06, 95% CI 0.005, 0.70, *P = *0.037)
5	Xue et al., [59]	Operation time is essential to save the patient's visual function; the earlier the operation time, the less damaged the optic nerve, and the greater the likelihood of recovery of visual function. Therefore, scholars are actively studying the optimal surgical timing of patients. However, no specific numerical data has been provided to quantify the impact of timing on visual outcomes

### Long-term outcomes and recurrence

Long-term follow-up data suggest that ONSF can provide sustained improvements in visual function. However, some patients may experience a recurrence of symptoms, necessitating additional procedures. For instance, Kelman et al. [24] reported significant long-term visual improvements with low reoperation rates, supporting the procedure's efficacy. In contrast, Knapp and Sampath [25] noted that 32% of patients experienced clinical recurrence post-ONSF, indicating the need for ongoing monitoring and potential additional interventions.

### Optic disc atrophy as a limiting factor in visual acuity recovery despite papilledema resolution after ONSF

Although ONSF is highly effective in reducing papilledema, the onset of optic atrophy before the surgery significantly limits the chances of notable visual recovery. This phenomenon is well recognized, with papilledema causing initial swelling of the optic nerve due to axoplasmic flow stasis. Without timely intervention, the persistent elevated pressure leads to progressive optic nerve damage, ultimately resulting in atrophy and permanent vision loss. While ONSF can alleviate mechanical compression and reduce optic disc edema, it cannot reverse the damage once optic atrophy has occurred, highlighting the critical need for early surgical intervention to prevent irreversible visual impairment [32, 59].

### Limitations

This meta-analysis is not devoid of limitations. First, the significant heterogeneity observed in the visual acuity and visual field outcomes suggests variability in surgical techniques, patient populations, and study designs across the included studies. This variability may influence the generalizability of the findings. Secondly, the included studies vary in methodological quality, and some lack detailed reporting on certain outcomes, leading to potential biases. Subsequently, the transconjunctival approach was the most commonly used surgical access method, while other approaches were infrequent, making it challenging to analyse each approach separately. Therefore, we created a separate group for transconjunctival access and another for other, non-transconjunctival approaches to facilitate meaningful comparisons. Additionally, the follow-up periods across the included studies were varied. To standardize the analysis, we selected the earliest follow-up time point available for each study.

### Comparison to the effectiveness of other methods of IIH treatment

In 2024, new meta-analyses evaluated the efficacy of other IIH treatment methods, though visual fields and visual acuity were not analysed separately. Andreão et al. did not find a significant difference in visual improvement between ventriculoperitoneal and lumboperitoneal shunts (OR 0.97, 95% CI: 0.26–3.62) [3]. Azzam et al. reported high improvement rates in papilledema (89%) and visual disturbances (88%) after venous stenting [4], while in our findings, 41.09% of patients showed improved visual acuity, slightly higher for the transconjunctival approach (43%). Visual field improvements were more significant, with a 76.34% pooled effect, favouring the transconjunctival approach (86%). Moreover, in our study optic disc swelling had a higher improvement rate (97%), advocating for increased effectiveness of ONSF over venous stenting. However, our findings on the improvement in optic disc swelling were affected by significant heterogeneity.

### Future research directions

Given the substantial heterogeneity observed in this meta-analysis, future research should aim to identify and control for factors contributing to this variability. Studies focusing on standardizing surgical techniques, improving patient selection criteria, and exploring the underlying mechanisms influencing surgical outcomes will be valuable. Additionally, addressing potential publication bias through more rigorous study designs and comprehensive reporting standards will enhance the reliability of future meta-analyses. Establishing a minimal clinical difference is also crucial to ensure that the observed effects are clinically significant and not merely statistically significant.

## Conclusions

Our meta-analysis corroborates the substantial body of evidence supporting the effectiveness of optic nerve sheath fenestration in improving and stabilizing visual outcomes for patients with IIH. While the procedure is generally safe, early intervention appears to be critical for optimal outcomes, particularly to avoid advanced ischemic damage. Notably, the transconjunctival approach to ONSF offers additional benefits, as it is associated with reduced tissue trauma, shorter operative times, and fewer postoperative complications, underscoring its potential as an efficient and safer alternative to more invasive techniques. Further research should also focus on understanding the mechanisms underlying the effectiveness of this surgery and on standardizing the methods for assessing surgical outcomes to facilitate more homogeneous meta-analyses in the future. Additionally, refining surgical techniques and identifying patient-specific factors that predict successful outcomes will further enhance the clinical utility of ONSF in managing IIH. This meta-analysis confirms the beneficial effects of surgical interventions on key visual outcomes while highlighting significant variability that warrants further investigation. By addressing these variations and potential biases, future research can contribute to optimizing surgical practices and improving patient-specific treatment strategies.

## Data Availability

No datasets were generated or analysed during the current study.
